# T1 mapping and major cardiovascular events in non‐ischaemic dilated cardiomyopathy: a systematic review and meta‐analysis

**DOI:** 10.1002/ehf2.15279

**Published:** 2025-04-25

**Authors:** Federico Marchini, Beatrice Dal Passo, Gianluca Campo, Elisabetta Tonet, Matteo Serenelli, Alberto Cossu, Serena Chiarello, Maria Lo Monaco, Erika Bertella, Rita Pavasini

**Affiliations:** ^1^ Cardiovascular Institute Azienda Ospedaliero Universitaria di Ferrara Ferrara Italy; ^2^ Division of Radiology Azienda Ospedaliero Universitaria di Ferrara Ferrara Italy; ^3^ Cardio Center Humanitas Gavazzeni Bergamo Italy

**Keywords:** Cardiovascular magnetic resonance, CMR, ECV, MACE, Native T1 mapping, Non‐ischaemic dilated cardiomyopathy

## Abstract

**Aims:**

The aim of this study is to investigate the prognostic role of T1 mapping techniques in predicting major adverse cardiovascular events (MACE) in patients affected by non‐ischaemic dilated cardiomyopathy (NIDCM) by performing a meta‐analysis of available studies.

**Methods and results:**

Data from 12 observational studies exploring the prognostic role of native T1 mapping and extracellular volume (ECV) were analysed with random effect generic inverse variance. The primary endpoint was MACE defined as a composite of heart failure or arrhythmic‐related events, expressed as hazard ratio (HR) with 95% confidence interval (CI). Secondary main outcomes were heart failure‐related events, arrhythmic‐related events, and weighted mean difference of native T1 mapping values or ECVs between patients with or without MACE. Overall, 4025 patients with NIDCM were included. The median follow‐up length was 22 (IQR 14–22) months. The primary outcome of MACE occurred in 610 patients with a pooled HR for native T1 mapping values of 1.07 (95% CI 1.04–1.09, *I* ^2^ 31.5%) and a pooled HR for ECV of 1.37 (95% CI 1.29–1.44, *I* ^2^ 0%). HF‐related events occurred in 492 patients, with a pooled HR for T1 mapping of 1.05 (95% CI 1.03–1.07, *I* ^2^ 1%) and a pooled HR for ECVs of 1.43 (95% CI 1.25–1.61, *I* ^2^ 63%). Arrhythmic‐related events occurred in 118 patients, with a pooled HR for T1 mapping values of 1.09 (95% CI 1.07–1.12, *I* ^2^ 0%). The weighted mean difference of native T1 mapping between patients with and without MACE was 30.91 (95% CI 18.45–43.16, *I* ^2^ 16.72%), while the mean difference of ECV was 4.52 (95% CI 2.78–6.26, *I* ^2^ 86%).

**Conclusions:**

In NIDCM patients, native T1 mapping and ECV were associated with increased risk of the composite primary endpoint of MACE and the secondary endpoint of heart failure and arrhythmic‐related events.

## Graphical abstract

In NIDCM patients, native T1 mapping and ECV were associated with increased risk of the composite primary endpoint of MACE and the secondary endpoint of heart failure and arrhythmic‐related events. Patients who experienced MACE had higher values of both T1 mapping and ECV.

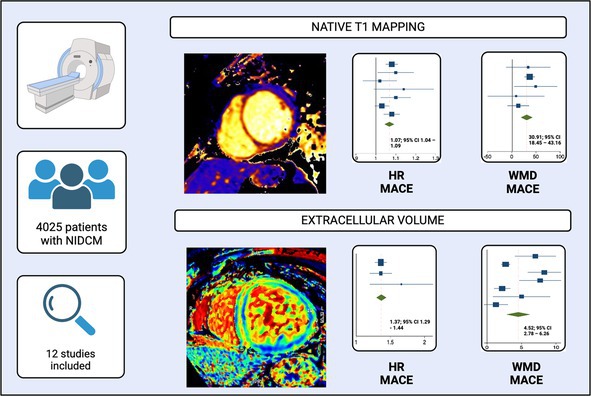

## Introduction

Non ischaemic dilated cardiomyopathy (NIDCM) is a primitive myocardial disorder characterized by left ventricular dilation and systolic dysfunction in the absence of significant coronary artery disease or anomalous loading conditions.^1^

NIDCM is one of the major causes of acute and chronic heart failure (HF) and the most frequent indication for heart transplantation.^2^ Despite advancements in diagnosis and treatment, risk stratification and prediction of adverse events remain a significant clinical challenge.

In recent years, cardiovascular magnetic resonance (CMR) has emerged as a fundamental imaging technique for diagnosis, management, and risk stratification of NIDCM. In fact, it represents not only the gold standard for a precise and reproducible evaluation of volumes and function, but it is also able to provide non‐invasive tissue characterization.

Macroscopic myocardial fibrosis assessed by late gadolinium enhancement (LGE) sequences has become of paramount importance in predicting cardiac remodelling, prognosis, and treatment options,^3^; however, the role of diffuse myocardial fibrosis is still under debate. Recently, parametric mapping techniques have emerged as a new way for a more advanced tissue characterization, and native T1 mapping and extracellular volume (ECV) fraction have become useful tools for the non‐invasive assessment of diffuse myocardial fibrosis.^4^, ^5^ In fact, NIDCM patients show significantly increased native myocardial T1 values and ECVs compared with the general population and recent studies suggested that ECV and native T1 mapping are independent prognostic predictors of mortality^6^; however, data from previous studies regarding their prognostic role are conflicting.

The aim of this meta‐analysis is to explore the role of CMR T1 mapping techniques in predicting major adverse cardiovascular events in NIDCM patients.

## Methods

We developed a systematic review and meta‐analysis following Preferred Reporting Items for Systematic reviews and Meta‐Analyses (PRISMA) amendment to the Quality of Reporting of Meta‐analyses (QUOROM) statement.^7^, ^8^, ^9^ The protocol of the present systematic review has been registered in PROSPERO with ID: CRD42024579651.

### Search strategy

Two expert cardiologists (F.M. and B.D.P) independently and systematically searched (MESH strategy) MEDLINE, Cochrane Library, Google Scholar, and Biomed Central for prospective studies whose objective was to evaluate the prognostic role of T1 mapping and ECV in patients with NIDCM. The terms searched were (((dilated cardiomyopathy) OR (non‐ischemic dilated cardiomyopathy)) AND ((T1 mapping) OR ((extracellular volume) OR (ECV)) OR ((cardiac MR) OR (cardiovascular magnetic resonance) OR (cardiac MRI) OR (cardiac magnetic resonance))) AND ((heart failure) OR (ventricular arrhythmias) OR ((cardiovascular death) OR (cardiac death)) OR (death) OR (prognosis) OR (outcomes))) NOT (meta‐analysis) NOT (review) NOT (case series) NOT (case report). Details of the search strategy are reported in the *Supporting Information*. The research was carried out in July 2024 and updated in January 2025.

### Selection criteria

The shortlisted studies were retrieved as full articles and appraised independently by two unblinded reviewers (F.M. and B.D.P.), with divergences solved after consensus, according to the following inclusion criteria: (i) English language, (ii) observational study, (iii) enrollment of NIDCM patients, (iv) execution of CMR with the acquisition of T1 mapping sequences, (v) availability of the individual outcome data of MACE (HF or arrhythmic‐related events), (vi) data published in peer‐reviewed journal and (vii) follow‐up length ≥1 year.

Exclusion criteria were (i) duplicate reports, (ii) failing to report additional or extended clinical outcomes, (iii) grey literature, (iv) abstract or posters, and (v) editorials and reviews.

### Data abstraction and endpoints

The reviewers (F.M. and B.D.P.) independently extracted data from full texts and published appendixes. The following information was retrieved: year of publication, journal, number of patients included, follow‐up length, inclusion and exclusion criteria, age, sex, cardiovascular risk factors, left ventricular indexed end‐diastolic volume, left ventricular ejection fraction, LGE presence, and scanner field strength. The primary outcome was major adverse cardiovascular events defined as a composite of HF or arrhythmic‐related events. Secondary outcomes were (i) HF‐related events, (ii) arrhythmic‐related events, and (iii) the weighted mean difference of native T1 mapping values or ECVs between NIDCM patients with or without MACE. Definitions of the study endpoints and CMR characteristics are detailed for each study in *Tables* *S1* *and* *S2*.

### Internal validity and quality appraisal

The quality of the included studies has been appraised by two unblinded reviewers (F.M. and R.P.) tested using pre‐specified electronic forms of MINORS criteria. Twelve methodological items were scored on a scale from 0 to 2: *not reported* (0 point), *reported but inadequate* (1 point), or *reported and adequate* (2 point). A minimum score of 10 and a maximum of 19 were achieved. No study was excluded based on this score (*Table* *S3*).

### Data analysis and synthesis

Continuous variables were reported as mean ± standard deviation or median (interquartile range). Categorical variables were expressed as number and percentage. Only studies reporting the effect size of the primary endpoint as HR with 95% confidence interval (CI) or the mean values of native T1 mapping and ECV in each study group (NIDCM patients with or without MACE) were included. We performed separate analyses according to T1 mapping technique (i.e. native T1 or ECV). For each outcome, the pooled event rate (ER), weighted mean difference, and HR with 95% CI were calculated. Standard errors were calculated by the formula: root squared (ER * (1 − ER)/sample size). Given that important differences existing between T1 sequences,^10^ the pooled univariate HR for primary and secondary endpoints was calculated including only studies that reported an HR for a standardized increase of 10 ms in native T1 mapping values and 3% in ECVs. For the evaluation of the pooled effect size, the HR for native T1 mapping calculated on mid‐SAX was used. The multivariate pooled HR was not assessed because of the high variability of multivariate models. For the analyses of ER, HR, and mean differences, DerSimonian and Laird random effects model were used with heterogeneity being taken from the inverse variance random‐effect model.^11^ Statistical heterogeneity was assessed using Cochran's *Q* test and *I* ^*2*^ statistics, which quantifies the proportion of total variation across studies that is due to heterogeneity rather than chance. A value of *I* ^*2*^ of 0–25% represents insignificant heterogeneity, 26–50% low heterogeneity, 51–75% moderate heterogeneity, and >75% high heterogeneity.^12^ Sensitivity analyses were also performed using the leave‐one‐out approach for the primary outcome of MACE. Subgroup analysis using the ANOVA test was performed to access the impact on the outcomes of left ventricular ejection fraction (LVEF) < 35%, left ventricular end‐diastolic volume indexed (LVEDVi) > 120.5 mL/m^2^,^13^ and the mean prevalence of LGE. Because of the small number of studies analysed for each effect size (<10), it was not possible to perform publication bias and meta‐regression analyses.^14^ Stata/SE version 16 (Stata Corp, College Station, TX, USA) and Prometa (Internovi, Cesena, Italy) softwares were used for statistical analyses.

## Results

### Search results and study selection

**Figure 1:**
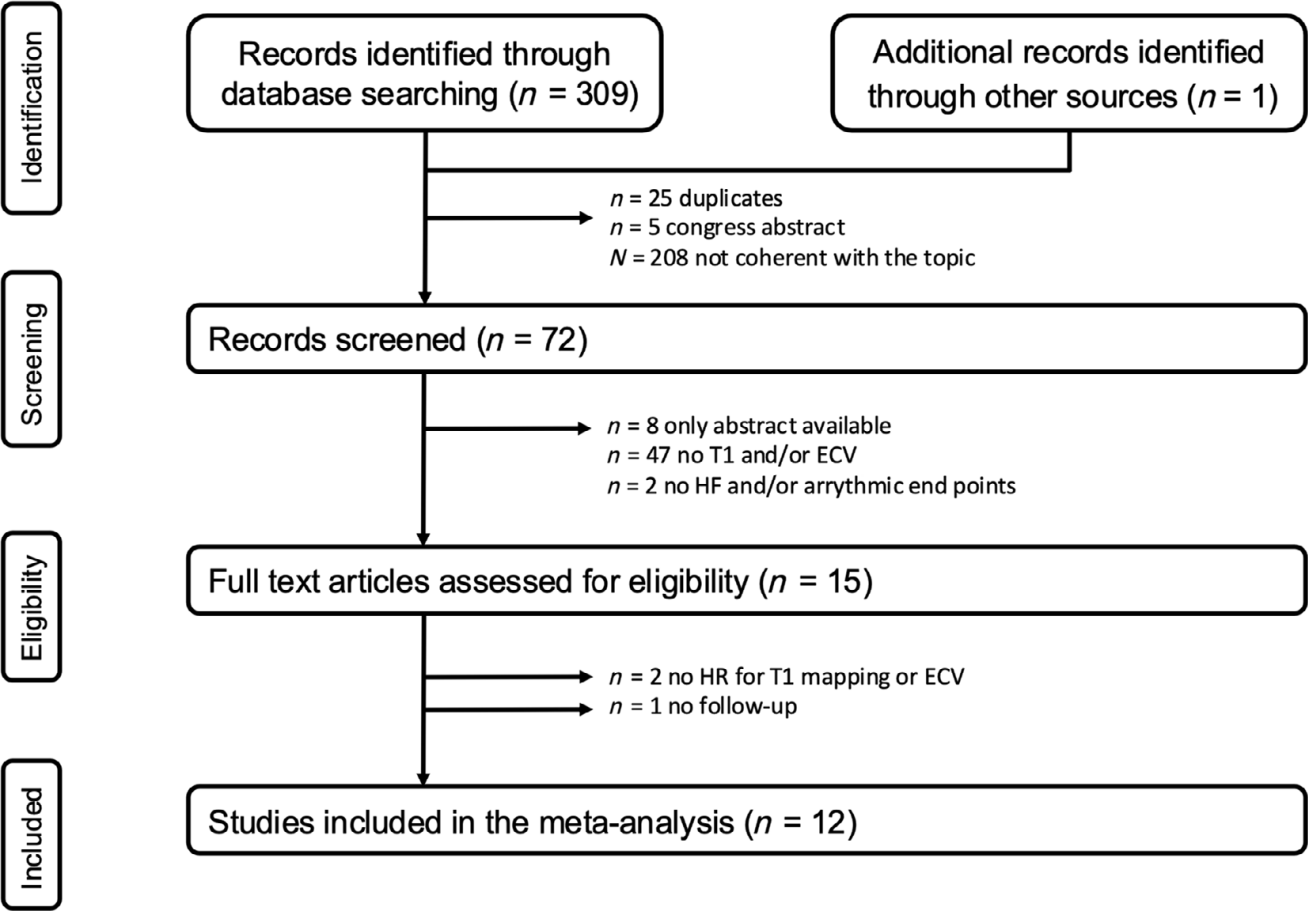
Outline of the search strategy.

The database search yielded 310 records (*Figure* *1*). After the first evaluation of title and abstract, 25 duplicates, five congress abstract, and 208 studies not related to the topic of this meta‐analysis were excluded. Between the 72 records screened, 47 were excluded due to the absence of data on T1 mapping techniques, two were excluded due to the lack of the desired outcome, and eight were excluded as only abstracts were available. As result, 15 studies were analysed as full text. Two studies were excluded because HR for T1 mapping or ECV was not available.^15^, ^16^ The study of Rubìs *et al*.^17^ was excluded for lack of follow‐up. Finally, 12 studies were included in the analysis.^6^, ^18^, ^19^, ^20^, ^21^, ^22^, ^23^, ^24^, ^25^, ^26^, ^27^, ^28^

Seven studies reported the HR for a standardized increase of 10 ms in native T1 mapping values,^6^, ^18^, ^23^, ^25^, ^26^, ^27^, ^28^ while three studies^20^, ^22^, ^24^ reported the HR for a standardized increase of 3% in ECVs. The studies of Lee *et al*.^18^ and Xu *et al*.^27^ reported the HR for both HF‐ and arrhythmic‐related events and the pooled HR for the composite outcome was calculated. In the study of Vita *et al*.,^20^ the reported HR for 10% increase in ECV values was converted in 3% increase using formula accepted in literature.^29^

Finally, for the calculation of the weighted mean difference, five studies reported the values of native T1 mapping between patients with and without MACE,^20^, ^22^, ^24^, ^26^, ^27^ and seven studies those for ECV.^19^, ^20^, ^21^, ^22^, ^24^, ^26^, ^27^

### Patients' characteristics

The twelve studies included 4,025 patients with NIDCM. The median follow‐up length was 22 (IQR 14–22) months (the minimum follow‐up was 12 months, the maximum 51 months). The mean age was 52 ± 5 years, and the mean proportion of female patients was 31%. About the main CMR characteristics, the mean LVEDVi was 145 mL/m^2^, mean LVEF was 32%, and the mean presence of LGE in the overall population was 50%. The main characteristics of the study population are detailed in *Table* *1*.

**Table 1:** Baseline characteristics of the patients.

First author, year	*N*	Age (y)	Female (%)	BMI (kg/m^2^)	Diabetes (%)	Hypertension (%)	Smoke (%)	Dyslipipidaemia (%)	AF (%)	HCT (%)	T1 mapping value (ms)	LVEF (%)	LGE presence (%)	LVEDVi (mL/m^2^)
Li *et al*., 2023	858	48 ± 15	30	24 ± 4	13	23	43	NA	18	NA	1307 ± 75	25.9 ± 12	44	175.8 ± 58.4
Puntman *et al*., 2016	637	50 (IQR 37–76)	38	27 (IQR 23–30)	24	48	28	30	8	43 (IQR 39–46)	997 (IQR 958–1056) [1.5 T] 1113 (IQR 1064–1157) [3.0 T]	47 (IQR 29–50)	27	109 ± 7
Vita *et al*., 2019	240	49 ± 16	38	28 ± 7	13	30	16	21	6	40 ± 4	1092 ± 127	43 ± 15	35	114 ± 13
Li *et al*., 2022	659	45 ± 15	24	21.6 ± 2.6	6.2	17.6	17.5	9.6	NA	NA	951 ± 55	29.5 ± 10.3	53.9	146.4 ± 58.7
Youn *et al*., 2017	117	51.9 ± 16.7	39.3	24.3 ± 4.2	18.8	38.5	NA	NA	NA	41.1 ± 6.5	1326.3 ± 91.1	24.9 ± 8.1	NA^a^	159.1 ± 52.4
Chen *et al*., 2018	46	46.7 ± 12.9	28	23.8 (IQR 21.7–27)	15	17	32	30	20	43.9 ± 5.9	1367 ± 79	20 (IQR 15–27,5)	80	201.7 ± 50.8
Nakamori *et al*., 2020	115	54 ± 15	23	28.8 ± 6.2	17	35	10	31	15	NA	1127 ± 44	30.7 ± 5.9	49	142.1 ± 41.6
Claridge et al, 2016	130	58.5 ± 15.91	21.3	NA	12.1	24.5	NA	NA	32.8	NA	1054 ± 7.15	NA	NA	NA
Cadour *et al*., 2023	225	57.5 ± 14.5	36.1	26.7 ± 5.2	14.7	33.5	38.6	26.4	6.6	41.9 ± 4.5	3.0 ± 2.3^b^	29.3 ± 9.7	51.8	145 ± 48
Kodama *et al*., 2020	60	61 ± 12	28	24 ± 4	30	52	NA	25	NA	NA	NA	37 ± 11	50	NA
Di Marco *et al*., 2022	703	59 (IQR 49–68)	34	NA	NA	NA	NA	NA	27	NA	55 (IQR 26–89)^c^	42 (IQR 32–48)	42	111 (91–134)
Xu *et al*., 2024 (MACE+)	54	48.2 ± 14.2	33	23.4 ± 3.9	14.8	9.3	NA	NA	NA	46 ± 5	1325.2 ± 71.1	22.2 ± 10.3	63.4	189.3 ± 53.7
Xu *et al*., 2024 (MACE−)	181	45.5 ± 14.1	33	24.8 ± 4.4	12.2	18.9	NA	NA	NA	45 ± 6	1311.8 ± 72.7	27.0 ± 11.2	38.8	160.4 ± 47.5

Values are expressed in mean ± standard deviation (DS) unless otherwise indicated.

AF, atrial fibrillation; BMI, body mass indexed; HCT, haematocrit; LGE, late gadolinium enhancement; LVEDVi, indexed left ventricular end‐diastolic volume; LVEF, left ventricular ejection fraction.

^a^ Not reported for overall population.

^b^ Mean native T1 Z‐score.

^c^ Mean T1 mid segments standardized as the difference between the observed mean mid‐T1 value and reported normal value.

### Primary outcome

**Figure 2:**
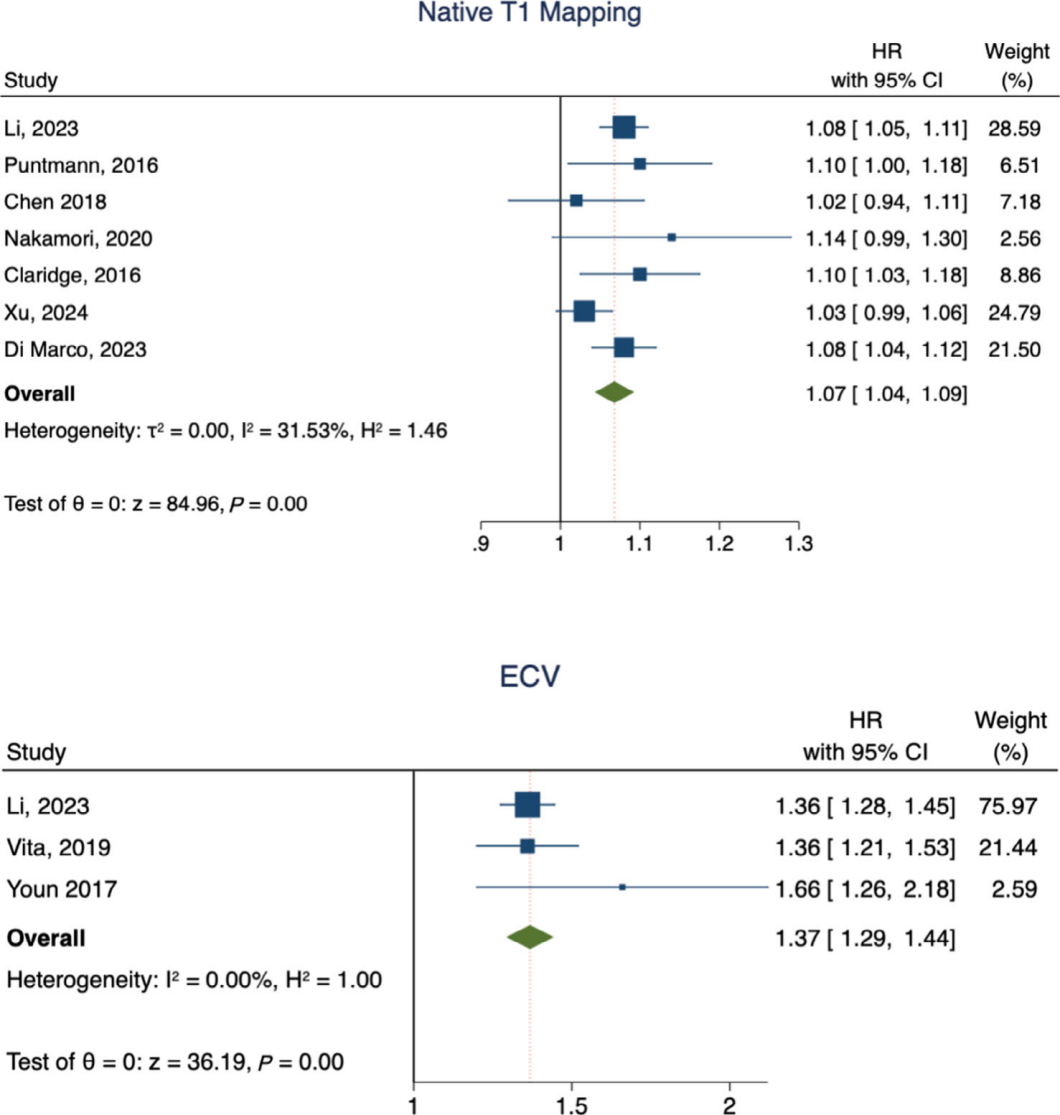
Summary plots for MACE. ECV: extracellular volume.

The primary outcome of MACE occurred in 610 patients. The pooled event rate was 16% (95% CI 12–20, *I* ^*2*^ 91.5%). The pooled HR for 10 ms increase in native T1 mapping values was 1.07 (95% CI 1.04–1.09, *I* ^*2*^ 31.5%), while the pooled HR for 3% increase in ECV values was 1.37 (95% CI 1.29–1.44, *I* ^*2*^ 0%) (*Figure* *2*). No difference was found according to LVEDVi and severely reduced LVEF in the subgroup analysis (*P* = 0.7 and *P* = 0.41, respectively; *Figure* *S1* and *Figure* *S2*.)

### Secondary outcomes

**Figure 3:**
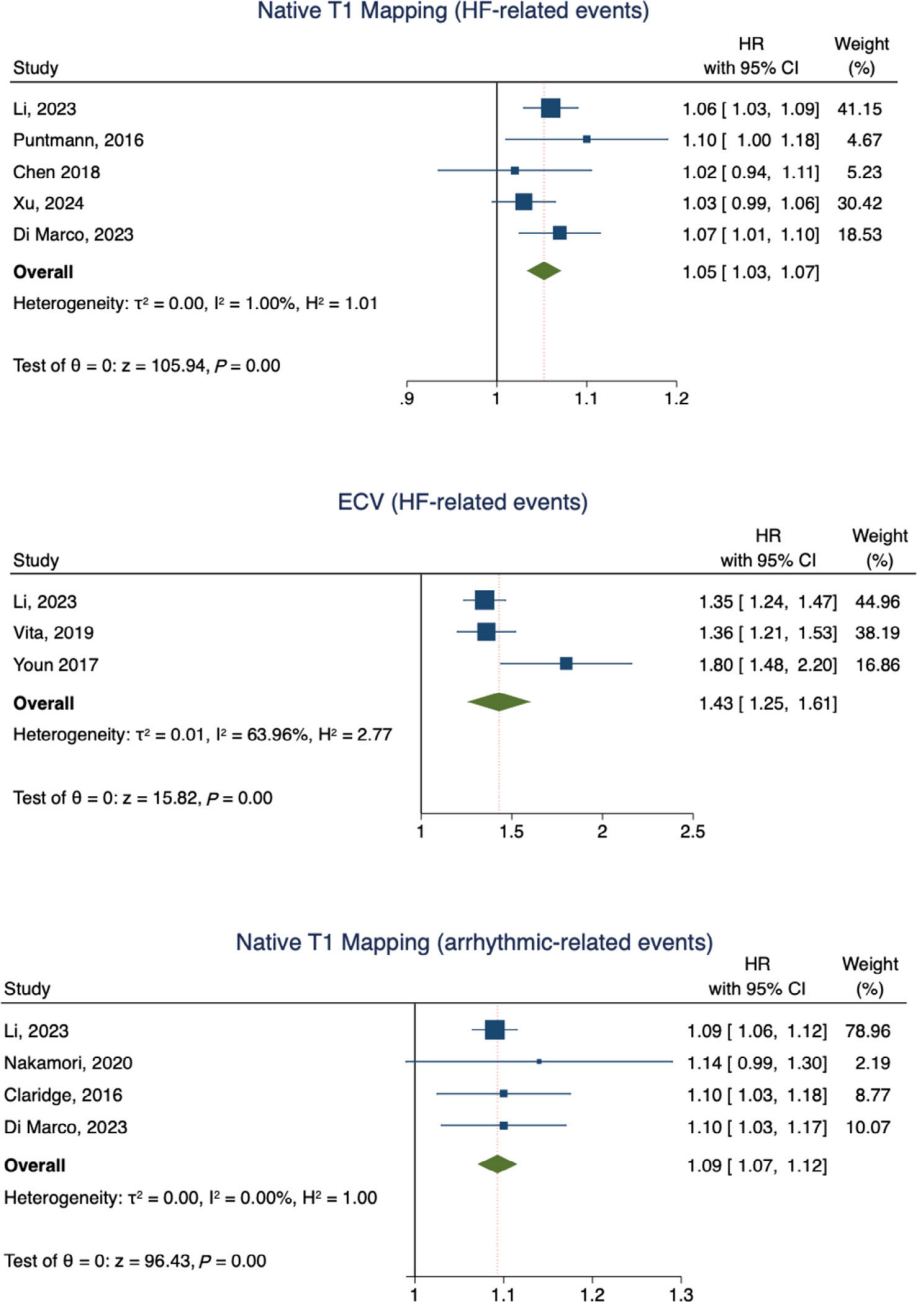
Summary plots for secondary endpoints. ECV, extracellular volume; HF, heart failure.

HF‐related events occurred in 492 patients (pooled event rate 14%, 95% CI 10–18, *I* ^*2*^ 92.3%) with a pooled HR for 10 ms increase in native T1 mapping of 1.05 (95% CI 1.03–1.07, *I* ^*2*^ 1%) and a pooled HR for 3% increase in ECVs of 1.43 (95% CI 1.25–1.61, *I* ^*2*^ 64%). Arrhythmic‐related events occurred in 118 patients (pooled event rate 7%, 95% CI 3–12, *I* ^*2*^ 93.7%) with a pooled HR for 10 ms increase in native T1 mapping values of 1.09 (95% CI 1.07–1.12, *I* ^*2*^ 0%) (*Figure* *3*). Finally, the weighted mean difference of native T1 mapping between patients with and without MACE was 30.91 (95% CI 18.45–43.16, *I* ^*2*^ 16.72%), while the mean weighted difference of ECV was 4.52 (95% CI 2.78–6.26, *I* ^*2*^ 86%) (*Figures* *S3* and *S4*). Subgroup analysis according to LGE prevalence and LVEF is available in *Figure* *S5*.

### Sensitivity analysis

Sensitivity analysis with the ‘leave‐one‐out approach’ showed that the results about MACE was confirmed also after removing data from one study at a time. These findings apply both for native T1 mapping and ECV (*Tables* *S4* and *S5*).

## Discussion

The present meta‐analysis aimed to investigate the prognostic role of CMR T1 mapping techniques in predicting major adverse cardiovascular events in patients with non‐ischemic dilated cardiomyopathy. The main findings are:

- Native T1 mapping and ECV are associated with increased risk of the composite primary endpoint of MACE and secondary outcome of HF and arrhythmic‐related events.
- NIDCM patients suffering from MACE showed higher values of both T1 mapping and ECV, resulting in a significant overall weight mean difference.

The results of the present meta‐analysis have important clinical implications, as they may improve risk stratification for NIDCM patients. In fact, after the DANISH trail failed to prove prognostic significance of reduced LVEF in guiding ICD implantation in primary prevention of NIDCM patients,^30^ new markers for high‐risk were needed. LVEF provides a global assessment of left ventricular systolic function, but it has limited correlation with the underlying disease that contributes to adverse events. Moreover, only a small portion of patients with severely reduced LVEF benefits from ICD in primary prevention of arrhythmic events, which may also occur in patients with normal or not severely reduced LVEF.^31^ The identification of novel prognostic stratification strategies is essential to target those patients who would derive substantial benefit from ICD implantation, while concurrently avoiding unnecessary procedures in individuals with a low risk of sudden cardiac death.

Myocardial fibrosis plays a pivotal role in the development of arrhythmic events and SCD. In the DERIVATE‐NIDCM registry, Guaricci *et al*. found that LGE in three or more myocardial segments was an independent predictor of both all‐cause mortality and major adverse arrhythmic cardiac events (MAACE); in this study, the authors developed a composite clinical and CMR‐based risk score that provided a net reclassification improvement of 63.7% for MAACE occurrence when added to the model based on standard of care evaluation.^13^ The recent ‘2023 ESC Guidelines for the management of cardiomyopathies’ remarked the importance of LGE in risk stratification of NIDCM without a genotype associated with high SCD risk and LVEF >35%.^32^

Mid‐wall replacement fibrosis identified by LGE is a powerful marker of adverse events in NIDCM; nevertheless, it is observed in approximately 30% of NIDCM patients and may not fully represent diffuse interstitial fibrosis.^33^, ^34^ Myocardial interstitial fibrosis represents a final common lesion caused by a variety of injuries caused by intrinsic or systemic risk factors, and it is strongly associated to both arrhythmic events and left ventricular dysfunction leading to the development of HF.^35^ In fact, alterations of the myocardial physical properties, cellular interactions, tissue architecture, and extracellular matrix reservoir functions are the main determinants of left ventricular dysfunction and propensity to arrhytmias.^36^

Parametric mapping techniques have recently emerged as a new way for a more advanced tissue characterization thanks to the quantification of T1, T2, and T2* relaxation times on pixel‐by‐pixel maps.^37^ The native T1 is an inherent tissue‐specific property that has been shown to be highly effective in distinguishing healthy myocardium from diffusely diseased tissue. In fact, since each tissue shows a characteristic range of normal T1 relaxation times at a particular field strength, any alteration may be indicative of disease. T1 mapping techniques are useful tools for the non‐invasive assessment of diffuse myocardial fibrosis,^4^ and several studies proved that NIDCM patients show significantly increased native myocardial T1 values and ECVs compared with the general population.^6^, ^38^ Our meta‐analysis provides additional evidence that patients with NDCM who develop major adverse cardiovascular events have higher native T1 mapping and ECV values. However, the results of ECV should be carefully analysed because of the high heterogeneity observed (*I* ^2^ 86%). Although we did not perform a meta regression analysis, the subgroup analysis showed that the high heterogeneity was mainly related to studies with high prevalence of LGE and reduced LVEF.

The prognostic role of T1 mapping techniques remains uncertain, as studies have yielded conflicting results regarding its association with HF‐ or arrhythmic‐related events. This may be in part related to the lack of standardization of T1 mapping techniques: in fact, an optimal global cut‐off is absent and the reported HR is influenced by methodological differences in T1 mapping calculation, such as the selection of ROI (septum vs. mid SAX) and the use of mean values or of a standardized increase. The meta‐analyses published to date are not able to fully resolve this pivotal clinical answer. In a recent work form Berdibekov *et al*.,^39^ the authors found that increase in both T1 mapping and ECV were associated to MACE; however, these results need to be carefully analysed due to the high degree of heterogeneity displayed. A previous meta‐analysis from Kiaos *et al*.^40^ proved that both ECV and native T1 mapping had a significant prognostic value for a composite endpoint point of morbidity and mortality; however, more solid results on the association with HF‐ and arrhythmic‐related events are needed due to the high clinical significance for daily practice. Finally, in the work of TAO *et al*.,^41^ the authors found that native T1 mapping and ECV were significantly higher in NIDCM patients compared to control, in NIDCM patients who experienced MACE, and in those who were low treatment responder. No difference was found between NIDCM patients with ventricular arrhythmias, probably due to the low statistical power within the small sample sizes in the three included studies. As far as we concern, this is the first meta‐analysis focusing not only on the mean difference of T1 mapping values and ECVs between patients with and without MACE but also on the specific outcomes of HF‐ and arrhythmic‐related events. In fact, our study proved that both native T1 mapping and ECV are related to the composite primary endpoint of MACE and to the secondary endpoints of HF‐ and arrhythmic‐related events. Moreover, by restricting our analysis to studies that reported a standardized 10 ms increase in T1 mapping values and a 3% increase in ECV values, we achieved low or insignificant heterogeneity for both primary and secondary endpoints of native T1 mapping and moderate heterogeneity for HF secondary endpoint of ECV (data confirmed also after sensitivity analyses).

Our results further support the compelling evidence that native T1 and ECV could be valuable supplementary tools for risk assessment in NIDCM patients, facilitating a more targeted surveillance and intervention for those at higher risk of developing arrhythmic events and HF.

## Limitations

The main limitation of our analyses is the small number of studies included, which may limit the generalizability of the findings and precludes the possibility of performing a meta‐regression analysis. In addition, no randomized clinical trials have been included, and the difference in follow‐up length, inclusion and exclusion criteria, and CMR T1 mapping technique calculation and values should be considered. Furthermore, only univariate pooled HR was calculated, making our ability to investigate an independent association between T1 mapping techniques and MACE limited.

Finally, the analysis may be limited by the lack of individual patient data (IPD), which restricts the possibility to perform more specific subgroup analyses and adjust for patient‐level covariates.

Future research should aim to address these limitations by incorporating IPD and employing more rigorous and standardized methodologies to enhance the validity and reliability of the findings.

## Conclusions

T1 mapping and ECV are predictors of MACE in NIDCM patients. These findings highlight the importance of CMR in risk stratification of NIDCM patients and may support the use of these sequences to guide clinical decision‐making.

## Conflict of interest

The authors have no relevant financial or non‐financial interests to disclose.

## Funding

The authors declare that no funds, grants, or other support were received during the preparation of this manuscript.

## Supporting information


**Table S1.** Study characteristics.
**Table S2.** CMR characteristics of the studies.
**Table S3.** MINORS criteria.
**Figure S1.** T1 mapping and MACE according to mean LVEF.
**Figure S2.** T1 mapping and MACE according to mean LVEDVi.
**Figure S3.** Mean weighted difference of T1 mapping.
**Figure S4.** Mean weighted difference of ECV.
**Figure S5.** Subgroup analysis for the mean difference of ECV according to LVEF and LGE prevalence.
**Table S4.** T1 mapping sensitivity analysis for MACE.
**Table S5.** ECV sensitivity analysis for MACE.

## Data Availability

All data relevant to the study are included in the article oruploaded as supporting information.
