# Supplementary material for: T1 mapping and major cardiovascular events in non‐ischaemic dilated cardiomyopathy: a systematic review and meta‐analysis
Source: ESC Heart Fail. 2025 Apr 25;12(4):2621–30. doi: 10.1002/ehf2.15279 (PMC12287780; doi:10.1002/ehf2.15279)
Supplement: Supplementary file 1 — Table S1. Study characteristics. Table S2. CMR characteristics of the studies. Table S3. MINORS criteria. Figure S1. T1 mapping and MACE according to mean LVEF. Figure S2. T1 mapping and MACE according to mean LVEDVi. Figure S3. Mean weighted difference of T1 mapping. Figure S4. Mean weighted difference of ECV. Figure S5. Subgroup analysis for the mean difference of ECV according to LVEF and LGE prevalence. Table S4. T1 mapping sensitivity analysis for MACE. Table S5. ECV sensitivity analysis for MACE. [file EHF2-12-2621-s001.docx]

**Supplementary Informations**

**T1 mapping and major cardiovascular events in non-ischemic dilated cardiomyopathy: a systematic review and meta-analysis**

- **Search strategy details page 2**
- **Study endpoints page 4**
- **Table 1s page 5**
- **Table 2s page 13**
- **Table 3s. page 15**
- **Figure 1s page 16**
- **Figure 2s page 17**
- **Figure 3s page 18**
- **Figure 4s page 18**
- **Figure 5s page 19**
- **Table 4s page 20**
- **Table 5s page 20**

**Search strategy details**

((((("cardiomyopathy, dilated"[MeSH Terms] OR ("cardiomyopathy"[All Fields] AND "dilated"[All Fields]) OR "dilated cardiomyopathy"[All Fields] OR ("dilated"[All Fields] AND "cardiomyopathy"[All Fields]) OR (("non ischaemic"[All Fields] OR "non ischemic"[All Fields]) AND ("cardiomyopathy, dilated"[MeSH Terms] OR ("cardiomyopathy"[All Fields] AND "dilated"[All Fields]) OR "dilated cardiomyopathy"[All Fields] OR ("dilated"[All Fields] AND "cardiomyopathy"[All Fields])))) AND (("T1"[All Fields] AND ("mapped"[All Fields] OR "mapping"[All Fields] OR "mappings"[All Fields])) OR (("extracellular"[All Fields] AND ("volum"[All Fields] OR "volume"[All Fields] OR "volumes"[All Fields] OR "voluming"[All Fields])) OR "ECV"[All Fields]) OR ((("cardiacs"[All Fields] OR "heart"[MeSH Terms] OR "heart"[All Fields] OR "cardiac"[All Fields]) AND ("musculoskelet regen"[Journal] OR "ment retard"[Journal] OR "magn reson gott"[Journal] OR "mr"[All Fields])) OR (("cardiovascular system"[MeSH Terms] OR ("cardiovascular"[All Fields] AND "system"[All Fields]) OR "cardiovascular system"[All Fields] OR "cardiovascular"[All Fields] OR "cardiovasculars"[All Fields]) AND ("magnetic resonance spectroscopy"[MeSH Terms] OR ("magnetic"[All Fields] AND "resonance"[All Fields] AND "spectroscopy"[All Fields]) OR "magnetic resonance spectroscopy"[All Fields] OR ("magnetic"[All Fields] AND "resonance"[All Fields]) OR "magnetic resonance"[All Fields])) OR (("cardiacs"[All Fields] OR "heart"[MeSH Terms] OR "heart"[All Fields] OR "cardiac"[All Fields]) AND ("magnetic resonance imaging"[MeSH Terms] OR ("magnetic"[All Fields] AND "resonance"[All Fields] AND "imaging"[All Fields]) OR "magnetic resonance imaging"[All Fields] OR "mri"[All Fields])) OR (("cardiacs"[All Fields] OR "heart"[MeSH Terms] OR "heart"[All Fields] OR "cardiac"[All Fields]) AND ("magnetic resonance spectroscopy"[MeSH Terms] OR ("magnetic"[All Fields] AND "resonance"[All Fields] AND "spectroscopy"[All Fields]) OR "magnetic resonance spectroscopy"[All Fields] OR ("magnetic"[All Fields] AND "resonance"[All Fields]) OR "magnetic resonance"[All Fields])))) AND ("heart failure"[MeSH Terms] OR ("heart"[All Fields] AND "failure"[All Fields]) OR "heart failure"[All Fields] OR (("heart ventricles"[MeSH Terms] OR ("heart"[All Fields] AND "ventricles"[All Fields]) OR "heart ventricles"[All Fields] OR "ventricular"[All Fields] OR "ventricularization"[All Fields] OR "ventricularized"[All Fields]) AND ("arrhythmia s"[All Fields] OR "arrhythmias, cardiac"[MeSH Terms] OR ("arrhythmias"[All Fields] AND "cardiac"[All Fields]) OR "cardiac arrhythmias"[All Fields] OR "arrhythmia"[All Fields] OR "arrhythmias"[All Fields])) OR ((("cardiovascular system"[MeSH Terms] OR ("cardiovascular"[All Fields] AND "system"[All Fields]) OR "cardiovascular system"[All Fields] OR "cardiovascular"[All Fields] OR "cardiovasculars"[All Fields]) AND ("death"[MeSH Terms] OR "death"[All Fields] OR "deaths"[All Fields])) OR ("death"[MeSH Terms] OR "death"[All Fields] OR ("cardiac"[All Fields] AND "death"[All Fields]) OR "cardiac death"[All Fields])) OR ("death"[MeSH Terms] OR "death"[All Fields] OR "deaths"[All Fields]) OR ("prognosis"[MeSH Terms] OR "prognosis"[All Fields] OR "prognoses"[All Fields]) OR ("outcome"[All Fields] OR "outcomes"[All Fields]))) NOT ("meta analysis"[Publication Type] OR "meta analysis as topic"[MeSH Terms] OR "meta analysis"[All Fields])) NOT ("review"[Publication Type] OR "review literature as topic"[MeSH Terms] OR "review"[All Fields])) NOT (("ieee int conf automation sci eng case"[Journal] OR "case phila"[Journal] OR "case"[All Fields]) AND ("serie"[All Fields] OR "series"[All Fields]))) NOT ("case reports"[Publication Type] OR "case report"[All Fields])

**Study Endpoints**

1. **Primary composite endpoint of MACE**

As reported in previous studies, MACE was defined as an HF- or arrhythmic related event [1, 2].

1. An HF-related event was defined as at least one between: HF death, heart transplant, HF hospitalization, left ventricle assist device implantation for advance HF.
2. An arrhythmic-related events event was defined as at least one between: sudden cardiac death, appropriate ICD therapy, sustained ventricular arrhythmias, resuscitation after cardiac arrest, ventricular fibrillation.
3. **Secondary endpoint:**
   1. HF-related events.
   2. Arrhythmic-related events.
   3. Weighted mean difference between NIDCM patients with or without MACE.

**Table 1s** Study characteristics

| First Author, Year | N | Mean follow-up  (months) | Study design | Inclusion criteria | Exclusion criteria | Heart failure related events  (n/%) | Arrhythmia related events  (n/%) | Primary end-point | Secondary end-point |
| --- | --- | --- | --- | --- | --- | --- | --- | --- | --- |
| Li at al, 2023 | 858 | 33 (IQR 20.4 – 51.6) | Prospective, single center | Non-ischemic DCM with LVEF <50% and left LVEDD >55 mm. | CHD; primary valvular disease; HCM; cardiac amyloidosis; ARCV; alcoholic cardiomyopathy; myocarditis; ischemic heart disease (history of coronary artery revascularization or MI, more than 50% stenosis of the coronary artery on angiography or cardiac CT, or an infarction pattern of LGE on CMR). Contraindications to contrast-enhanced CMR, inadequate image quality, arrythmia, loss to follow-up or an unwillingness to sign the informed consent. | 97/11 | 70/8 | Sudden cardiac death related events: sudden cardiac death, appropriate ICD shock, resuscitation after cardiac arrest. | HF related events: HF death and heart transplant.  Composite end points, including cardiovascular death, heart transplant, appropriate ICD shock, resuscitation after cardiac arrest. |
| Puntman et al, 2016 | 637 | 22 (IQR 19-25) | Prospective, multicenter | NICDM with diagnosis confirmed by CMR study based on increased LV end-diastolic volume indexed to body surface area and reduced LVEF. | Ischemic heart disease, (defined as significant documented CAD, previous coronary revascularization, previous history of MI, or evidence of ischemic type LGE, or inducible ischemia on stress testing). Amyloidosis, iron accumulation, lipid-storage disease, HCM or ARVC or myocardial inflammation. Primary valvular heart disease. Contraindications to CMR (implantable devices, cerebral aneurysm clips, cochlear implants, severe claustrophobia), history of renal disease (GFR <30 ml/min/1.73 m2), unable to receive gadolinium contrast agent, and inability to provide informed consent. | 62/9 | NA | Death from any cause. | HF composite end-point, including HF death or unplanned HF hospitalization. |
| Vita et al, 2019 | 240 | 45.6 ±19.2 | Prospective, single center | All of the following  1) CMR referral primarily for assessing for etiology of cardiomyopathy;  2) symptoms or signs consistent of HF as identified by the referring physician;  3) LVEF <60%;  4) LVEDV indexed >80 ml/m2. | CAD (angiography or history or imaging evidence of MI or ischemia. Cardiac amyloidosis, cardiac sarcoidosis, HCM, Chagas disease. Moderate-to-severe valvulopathy; Absolute contraindications to performing CMR. | 36/15 | NA | MACE based on first HF related outcomes: death from any cause or heart failure decompensation requiring hospitalization. |  |
| Li et al, 2022 | 659 | 66.3 ± 20.9 | Retrospective, single center | DCM confirmed on CMR using the World Health Organization/ International Society and Federation of Cardiology definition of DCM, 1) LVEF <45%  2) LVEDV >2 SDs from normal according to normograms corrected by body surface area and age. | Ischemic heart disease (documented by coronary angiography, perfusion imaging, or medical records), infarct pattern of LGE on CMR studies, and/or ACS or coronary revascularization during follow-up)  Acute or sub-acute myocarditis, HCM, moderate-to-severe valvular disease, infiltrative disease, and/or other etiologies that might result in secondary DCM. | 122/18 | NA | Cardiac-related death (including HF death, sudden cardiac death) and heart transplant. | Hospitalization for HF, ventricular arrhythmias, ICD or cardiac resynchronization therapy implantation. |
| Youn et al, 2017 | 117 | 11,2 (IQR 7.8 – 21.9) | Prospective, single center | Patients with reduced LVEF ≤40% on CMR without any history of significant coronary artery disease and left ventricular chamber dilatation (enlarged LVEDD on short-axis view (≥6 cm)). | Myocarditis; newly diagnosed significant CAD; incomplete data sets. | 19/16 | NA | Cardiovascular death, rehospitalization due to HF, and heart transplant. |  |
| Chen et al, 2018 | 46 | 13 (IQR 7-13) | Prospective single center | Confirmed diagnosis of severe DCM (> 18 years old) with increased LVEDV index compared with the reference range; and LVEF < 35% in CMR. | Significant CAD (defined as ≥ 50% luminal stenosis), previous coronary revascularization, or MI. Valvular disease; CHD. GFR < 30 mL/min/1.73 m2 or implanted devices. | 9/19 | NA | Cardiac death, heart transplant, hospitalization due to cardiovascular events. |  |
| Nakamori et al, 2020 | 115 | 24 (IQR 13 - 37) | Prospective, single center | NICM who were scheduled to receive a primary ICD or undergo electrophysiology study, or both, or ablation. | Presence of any epicardial coronary artery dimeter stenosis >70%, a history of MI, or a subendocardium based LGE pattern; persistent AF; any contraindications to CMR; patients under the age of 18 | 13/11 | NA | Appropriate ICD therapy and sudden cardiac death. |  |
| Claridge et al, 2016 | 130 | 31 (IQR 22-39) | Prospective, single center | Patients undergoing ICD implantation for primary and secondary prevention. |  | NA | 18/14 | Appropriate ICD therapy for VT or ventricular fibrillation. | Composite of appropriate ICD therapy or death. |
| Cadour et al, 2023 | 225 | 23,9 (IQR 18.7 – 24.2) | Prospective, multicenter | NIDCM with typical symptoms of HF at the time of diagno- sis and an LVEF<45% with a LVEDV >90 ml/m2 measured by echocardiography | DCM was caused by hypertension, ischemic or valvular disease or HCM. Contraindications to CMR (claustrophobia, implantable devices, former metallic cardiac valves and non-CMR compatible vascular clips) or a history of renal disease (GFR <30 ml/min/1.73 m2). Patients with hepatic insufficiency; bone metabolism abnormalities; or unstable, nontreated or acute HF during the past month. | 42/19 | 16/7 | Heart failure-related events, including heart failure death, heart failure hospitalizations, heart transplant, left ventricular assist device implantation for advanced heart failure. Arrhythmia-related events, including: sudden death, sustained ventricular tachycardia, appropriate ICD shocks, resuscitated cardiac arrest, ventricular fibrillation. |  |
| Kodama e al, 2020 | 60 | 12 | Retrospective, single center | NIDCM patients with a LVEF of < 50% and no significant CAD on coronary angiography. | Severe valvular heart disease, GFR <30 ml/min/1.73 m2, contraindication to CMR (claustrophobia or after a metallic hazard implantation). | 7/12 | NA | Cardiac death; recurrent hospitalization due to HF. |  |
| Di Marco et al, 2022 | 703 | 21 | Prospective, single center, registry | NICM with LV systolic dysfunction in the absence of abnormal loading conditions or significant coronary artery disease (>70% luminal stenosis in a major coronary artery or >50% in the left main coronary artery). Systolic dysfunction was considered as reduced LVEF, as compared to published age- and sex-specific reference values. | Valvular heart disease (moderate or severe primary valvular dysfunction), previous MI or sub-endocardial LGE, infiltrative myocardial disease, sarcoidosis, HCM, CHD and probable or definite ARVC. | NA | 14/2 | Combined arrhythmic endpoint comprising sustained VT, appropriate ICD therapies (either anti-tachycardia pacing or shock), resuscitated cardiac arrest or sudden death. | Combined HF endpoint, including death due to end-stage HF, heart transplant, left ventricular assist device implant or HF hospitalization. |
| Xu et al, 2024 | 235 | 15.3  (IQR 12.5 - 19.2) | Prospective and consecutive registry, single center | DCM patients diagnosed as follows: reduced LVEF (< 45%) on CMR; LVEDD >55 mm. | Significant coronary artery disease (> 50% luminal stenosis) or evidence of an ischemic LGE pattern; primary mitral or aortic valve disease; myocarditis or hypertensive heart disease, end-stage HCM, tachycardia-induced cardiomyopathy, and infiltrative diseases; patient unwillingness to provide informed consent or undergo follow-up CMR examinations; contraindications to CMR examination or poor image quality. | 48/20.4 | 6/2.6 | MACE composite endpoint, comprising HF, sudden cardiac death, and HF readmission. |  |

ICD = implantable cardioverter defibrillator. MACE = major adverse cardiovascular events. LVEF = left ventricular ejection fraction. LVEDD = left ventricular end diastolic diameter. CHD = congenital heart disease. HCM = hypertrophic cardiomyopathy. ARCV = arrhythmogenic right ventricular cardiomyopathy. MI = myocardial infarction. CT = computed tomography. LGE = late gadolinium enhancement. CMR = cardiac magnetic resonance. HF = heart failure. LVEDV = left ventricular end diastolic volume. GFR = glomerular filtration rate. CAD = coronary artery disease. ACS = acute coronary syndrome. AF = atrial fibrillation. VT = ventricular tachycardia.

**Table 2s** CMR characteristics of the studies

| First Author, Year | CMR Scan | Field Strenght | T1 mapping sequences | Flip angle | Acquisition scheme | Contrast agent |
| --- | --- | --- | --- | --- | --- | --- |
| Li at al, 2023 | MAGNETOM Trio or Skyra; Siemens Health- care | 3.0-T | MOLLI | 50° | NA | Magnevist, Bayer Healthcare Pharmaceuticals |
| Puntman et al, 2016 | Achieva or higher, Philips Healthcare, Best, The Netherlands | 1.5-T or 3.0-T | MOLLI | 60° | 3(2)3(2)5 | Gadovist, Bayer Schering Pharma AG |
| Vita et al, 2019 | Tim Trio, Siemens, Erlangen, Germany | 3.0-T | MOLLI | 10° | NA | Magnevist, Bayer HealthCare Pharmaceuticals |
| Li et al, 2022 | Magnetom Avanto, Siemens Healthineers | 1.5-T | MOLLI | 85°- 65° | 5(3)3 | Magnevist, Bayer Healthcare Pharmaceuticals |
| Youn et al, 2017 | Magnetom Trio Tim; Siemens AG Healthcare Sector, Erlangen, Germany | 3.0-T | MOLLI | 35° | NA | Gadovist, Bayer Schering Pharma AG |
| Chen et al, 2018 | Ingenia, Philips Medical Systems, Best, the Netherlands | 3.0-T | MOLLI | 20° | 5(3)3 (pre-contrast)  4s(1s)3s(1s)2s  (post-contrast) | Magnevist, Bayer Healthcare Pharmaceuticals |
| Nakamori et al, 2020 | Achieva, Philips Medical Systems, Best, the Netherlands | 1.5-T | MOLLI | 70° | NA | MultiHance, Bracco Imaging SpA |
| Claridge et al, 2016 | Philips Healthcare, Best, The Netherlands | 1.5-T | MOLLI | 50° | NA | Gadovist, Bayer Schering Pharma AG |
| Cadour et al, 2023 | Siemens | 1.5-T or 3-T | MOLLI | 35° | 5(3)3 (pre-contrast)  4(1)3(1)2 (post constrast) | Dotarem, Guerbet, |
| Kodama e al, 2020 | Achieva; Philips Healthcare, Best, The Netherlands | 1.5-T | MOLLI | 35° | 5(3)3 | Gadolinium (not specified) |
| Di Marco et al, 2022 | 1.5 T Avanto or 3 T Skyra scanner (Siemens, Erlangen, Germany) | 1.5-T or 3-T | MOLLI | 35° | 5b(3b)3b (pre-contrast)  4b(1b)3b(1b)2b (post constrast) | Gadoterate meglumine, Dotarem, Guerbet |
| Xu et al, 2024 | MAGNETOM Trio or Skyra, Siemens Healthineers, Erlangen, Germany | 3-T | MOLLI | 35° | 5b(3b)3b pre-contrast)  4b(1b)3b(1b)2b(post constrast) | Magnevist; Bayer Schering Pharma, Berlin, Germany |

**MINORS**

1. A clearly stated aim
2. Inclusion of consecutive patients
3. Prospective collection of data
4. Endpoints appropriate to the aim of the study
5. Unbiased assessment of the study endpoint
6. Follow-up period appropriate to the aim of the study
7. Loss to follow up less than 5%
8. Prospective calculation of the study size

*Additional criteria in the case of comparative study*

1. An adequate control group
2. Contemporary groups
3. Baseline equivalence of groups
4. Adequate statistical analyses

Every item use "Not reported (0 point)", "Reported but inadequate (1 point), or "Reported and adequate (2 point)" to judge. The global ideal score being 16 for non-comparative studies and 24 for comparative studies.

**Table 3s.** MINORS criteria

| **References** | Q1 | Q2 | Q3 | Q4 | Q5 | Q6 | Q7 | Q8 | Q9 | Q10 | Q11 | Q12 | overall |
| --- | --- | --- | --- | --- | --- | --- | --- | --- | --- | --- | --- | --- | --- |
| Yangjie Li et al | 2 | 2 | 2 | 2 | 2 | 2 | 2 | 0 | na | na | na | na | 14 |
| Valentina O. Puntmann et al | 2 | 2 | 2 | 2 | 0 | 2 | 2 | 2 | na | na | na | na | 14 |
| Farah Cadour et al | 2 | 2 | 2 | 2 | 2 | 2 | 2 | 0 | na | na | na | na | 14 |
| Tomas Vita et al | 2 | 2 | 2 | 2 | 0 | 2 | 2 | 0 | na | na | na | na | 12 |
| Sho Kodama et al | 2 | 0 | 2 | 2 | 0 | 2 | 2 | 0 | na | na | na | na | 10 |
| Shuang Li et al | 2 | 2 | 2 | 2 | 0 | 2 | 2 | 0 | na | na | na | na | 12 |
| Rui Chen et al | 2 | 0 | 2 | 2 | 0 | 2 | 2 | 0 | 2 | 2 | 2 | 2 | 18 |
| Shiro Nakamori et al | 2 | 0 | 2 | 2 | 2 | 2 | 2 | 0 | 2 | 2 | 1 | 2 | 19 |
| Jong-Chan Youn et al | 2 | 2 | 2 | 2 | 0 | 2 | 2 | 0 | 2 | 2 | 1 | 2 | 19 |
| Simon Claridge et al | 2 | 2 | 2 | 2 | 2 | 2 | 2 | 0 | na | na | na | na | 14 |
| Di Marco et al | 2 | 2 | 2 | 2 | 2 | 2 | 2 | 0 | na | na | na | na | 14 |
| Xu et al | 2 | 2 | 2 | 2 | 2 | 2 | 2 | 0 | na | na | na | na | 14 |

**Figure 1s.** T1 mapping and MACE according to mean LVEF.

*LVEF: left ventricular ejection fraction*

**Figure 2s.** T1 mapping and MACE according to mean LVEDVi.

*LVEDVi: left ventricular end-diastolic volume indexed*

**Figure 3s.** Mean weighted difference of T1 mapping

*MACE: major adverse cardiovascular events*

**Figure 4s.** Mean weighted difference of ECV.

*MACE: major adverse cardiovascular events*

**Figure 5s.** Subgroup analysis for the mean difference of ECV according to LVEF and LGE prevalence
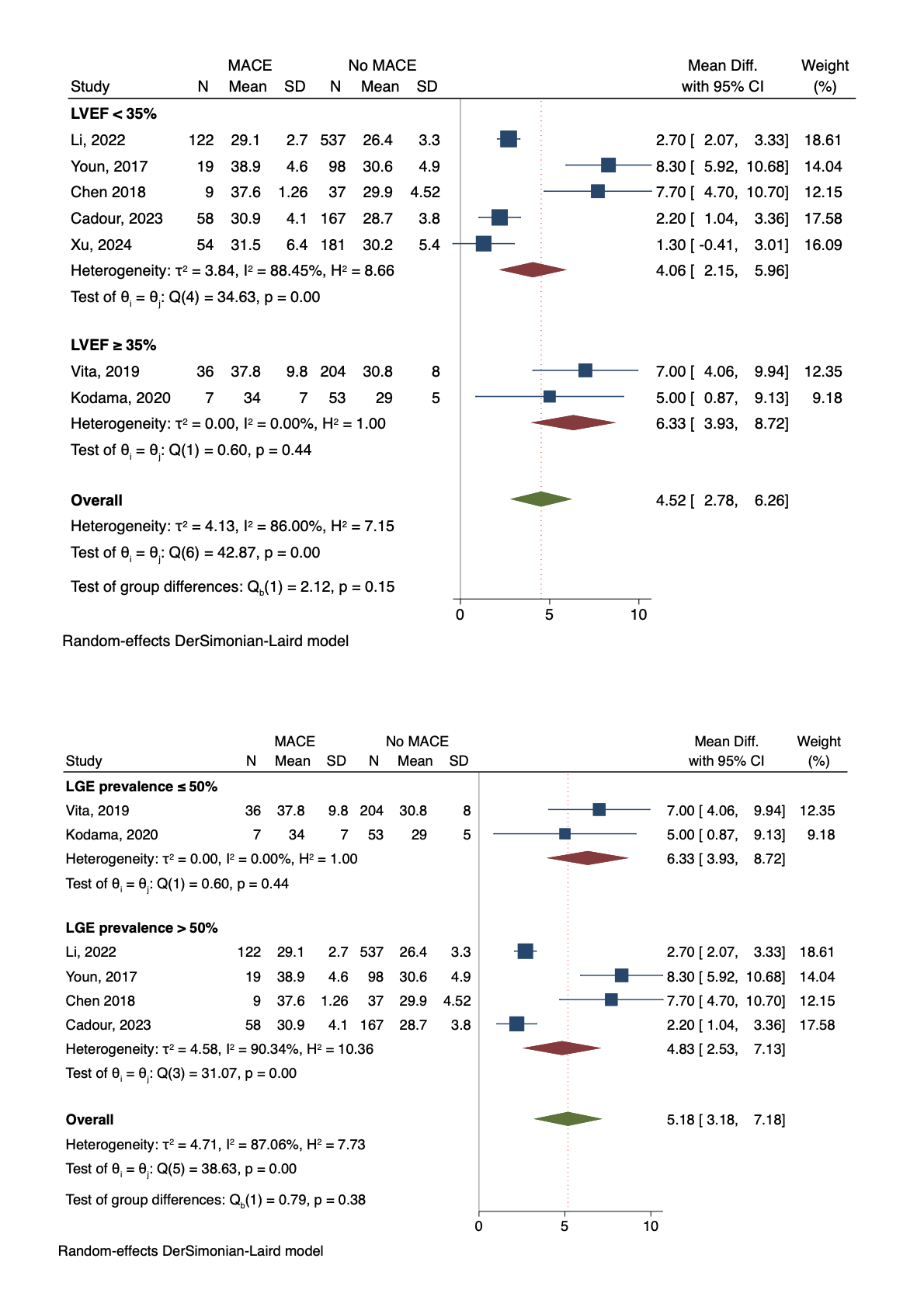


*MACE: major adverse cardiovascular events; LVEF: left ventricular ejection fraction; LVEDVi: left ventricular end-diastolic volume indexed*

**Table 4s** T1 mapping sensitivity analysis for MACE

| Study removed | HR | 95% CI | p value for overall size effect |
| --- | --- | --- | --- |
|  |  |  |  |
| Li, 2023 | 1.06 | 1.03 – 1.19 | < 0.001 |
| Puntmann, 2016 | 1.06 | 1.03 - 109 | < 0.001 |
| Chen 2018 | 1.07 | 1.04 – 1.19 | < 0.001 |
| Nakamori, 2020 | 1.06 | 1.04 – 1.09 | < 0.001 |
| Claridge, 2016 | 1.06 | 1.03 – 1.09 | < 0.001 |
| Xu, 2024 | 1.08 | 1.05 - 1.10 | < 0.001 |
| Di Marco, 2022 | 1.06 | 1.03 – 1.09 | < 0.001 |

**Table 5s.** ECV sensitivity analysis for MACE

| Study removed | HR | 95% CI | p value for overall size effect |
| --- | --- | --- | --- |
|  |  |  |  |
| Li, 2023 | 1.39 | 1.24 – 1.54 | < 0,001 |
| Vita, 2019 | 1.37 | 1.28 – 1.45 | < 0,001 |
| Youn, 2017 | 1.36 | 1.28 – 1.43 | < 0,001 |

**REFERENCES**

1. Cadour F, Quemeneur M, Biere L, et al (2023) Prognostic value of cardiovascular magnetic resonance T1 mapping and extracellular volume fraction in nonischemic dilated cardiomyopathy. Journal of Cardiovascular Magnetic Resonance 25:7. https://doi.org/10.1186/s12968-023-00919-y

2. Vita T, Gräni C, Abbasi SA, et al (2019) Comparing CMR Mapping Methods and Myocardial Patterns Toward Heart Failure Outcomes in Nonischemic Dilated Cardiomyopathy. JACC Cardiovasc Imaging 12:1659–1669. https://doi.org/10.1016/j.jcmg.2018.08.021
